# Ask the people: developing guidelines for genomic research with Aboriginal and Torres Strait Islander peoples

**DOI:** 10.1136/bmjgh-2021-007259

**Published:** 2021-11-03

**Authors:** Sid Kaladharan, Miranda E Vidgen, John V Pearson, Victoria K Donoghue, David C Whiteman, Nicola Waddell, Gregory Pratt, the GenetiQs Study, Greg Pratt

**Affiliations:** 1 Aboriginal and Torres Strait Islander Health, QIMR Berghofer Medical Research Institute, Herston, Queensland, Australia; 2 Nambour General Hospital, Sunshine Coast Hospital and Health Service, Nambour, Queensland, Australia; 3 Medical Genomics, QIMR Berghofer Medical Research Institute, Herston, Queensland, Australia; 4 Genome Informatics, QIMR Berghofer Medical Research Institute, Herston, Queensland, Australia; 5 Queensland Aboriginal and Islander Health Council, Brisbane, Queensland, Australia; 6 Cancer Control, QIMR Berghofer Medical Research Institute, Herston, Queensland, Australia

**Keywords:** public health

## Abstract

In health and medical research, guidelines are a set of statements and recommendations, whereby experts or stakeholders assess published literature to generate practical advice for a specific audience. This emphasis on guidelines development with expert consultation and published literature is not practical or inclusive when working in disciplines with minimal data and addressing issues that concern under-represented communities. Here we describe the process used for developing guidelines for the conduct of genomic research projects in partnership with Aboriginal and Torres Strait Islander peoples. A new technology with individual and community level ethical and social implications, and First Nations peoples with cultural and community expectations for research. We developed the guidelines through a consultation process that used participatory action research to engage with various stakeholders during multiple rounds of tailored activities. The end product, ‘Genomic Partnerships: Guidelines for Genomics Research with Aboriginal and Torres Strait Islander peoples of Queensland’ reflects the needs of the end-users and perspectives of the Aboriginal and Torres Strait Islander peoples, communities and organisations that participated. Through this process, we have identified recommendations for developing guidelines with other under-represented communities.

**Summary box**

- Health and medical research has a problematic history of working with under-represented communities.
- Researchers look to policies, standards and guidelines for advice when engaging these communities.
- Current practices in guidelines development are not practical or inclusive when working in disciplines with minimal data and addressing issues that concern under-represented communities.
- Here we describe a process used for developing guidelines that reflect the expectations and perspectives of under-represented communities.
- This paper provides details of the consultative process used and recommendations based on our experiences working with Aboriginal and Torres Strait Islander peoples when developing guidelines for genomic research.

## Introduction

Community and consumer engagement is critical for researchers undertaking health and medical research, with many countries mandating this as part of good research practice.^1–3^ Research that intends to affect specific communities or that relates to specialised fields of research, should consult and engage with those communities and consumers.^4^ When research is designed and conducted without the perspective or input of the community, research can perpetuate negative experiences, including exploitation, discrimination and cultural insensitivity.^5 6^

First Nations peoples are the historical and continuing custodians of lands and resources that are now populated, in the majority by people of different cultures or ethnic origins.^7^ In Australia, the involvement of Aboriginal and Torres Strait Islander peoples in research decision-making and practice is not only a regulatory expectation,^8 9^ but an expectation of communities to realise self-determination.^5 10^ Like First Nations peoples of other countries, Aboriginal and Torres Strait Islander peoples of Australia have considerable experience of Western health and medical research practices but without significant improvements to their social determinants of health.^5^

Genomics is a field of research that can influence health service delivery, and as such, genomic initiatives have been established globally to implement this new technology into routine patient care.^11 12^ Unlike, many clinical measurements that are limited to one aspect of a person at a single point in time, a person’s genome remains unchanged across their whole lives. Since a person’s genome is inherited from their biological parents, having a person’s genomic information gives insight into that person now and into the future, as well as insights into everyone who is biologically related to them. Genomic information is both deeply personal and broadly revealing. As such, genomics has the potential for significant social and ethical harm due to the types of information that is generated and the implications for its use.^13^ For these reasons, human research ethics guidelines have special considerations for genomics research.^14–17^

To date, very few genomic research projects have involved Aboriginal and Torres Strait Islander peoples.^18–21^ First Nations peoples’ internationally have raised concerns about the social implications of genomics research if trust, accountability and equity are not central to the interactions between the researchers and the community.^22^ In Australia, there have been consultations about the ethical implications of genomic research involving Aboriginal and Torres Strait Islander peoples.^23–25^ Some countries have recognised the special ethical implications of genomics research for First Nations peoples and have worked with these groups to develop guidelines and frameworks.^26 27^ However, Australia’s ethical guidelines lack specific advice on how to conduct genomic research involving Aboriginal and Torres Strait Islander peoples.^28^

In health and medical research, guidelines are typically a set of recommendations, ideally based on evidence and reflective practice or alternatively experience or professional opinion. They are generally informed by expert stakeholders’ assessment of published literature.^29 30^ Guideline development varies widely depending on the topic, intended audience and how the literature is assessed.^30^ The process of assessment can include public and consumer consultation, but often focuses on assessment of literature and advice from a group of technical specialists.^29^ Where there is a lack of evidence for sensitive or controversial topics, experiential contributions of lived experiences from community and stakeholders need to be sought to inform technical, ethical and legal discussions.^30^

Here we describe the consultation process used by the authors to develop a guide for researchers when they propose to work with Aboriginal and Torres Strait Islander peoples in genomic research. We intend for this work to be a model for consumer engagement to co-designing guidelines in areas where there is a lack of evidence-base or practice to draw from, or under-represented communities are affected by recommendations.

## Project overview

### Context of the project

The project brought together stakeholders (table 1) to inform the development of a guidelines document: ‘Genomic Partnerships: Guidelines for genomic research with Aboriginal and Torres Strait Islander peoples of Queensland’ (referred to as *Genomic Partnerships*).^31^ The project aimed to determine preferred practice for genomics research involving Aboriginal and Torres Strait Islander participants based on consultation. The target audience for the guidelines are primarily researchers who do not identify as Aboriginal and Torres Strait Islander or are not experienced with research involving this cohort of population. The guidelines needed to be accessible to this audience and align with national expectations for research practice. However, it was critically important to the credibility and acceptance of *Genomic Partnerships* that the guidelines reflected Aboriginal and Torres Strait Islander perspectives and expectations.

**Table 1:** Stakeholder groups involved in consultation for Genomic Partnerships

Term	Summary
Group 1	Researchers (particularly those with genomics experience) with limited or no experience working with Aboriginal and Torres Strait Islander peoples.
Group 2	Professionals with experience in research, research ethics, policy or health service delivery involving Aboriginal and Torres Strait Islander peoples, including clinicians and those who identify as Aboriginal and Torres Strait Islander.
Group 3	Aboriginal and Torres Strait Islander community members and health consumers.

**Figure 1:**
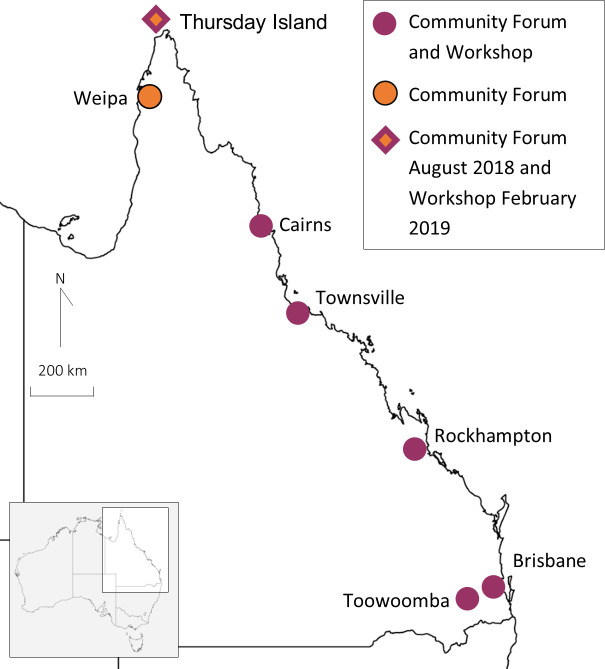
Map of Queensland with locations of workshops and community forums.

The consultation to develop *Genomic Partnerships* occurred in the Australian state of Queensland and was delivered in English. Queensland has a population of 4.8 million people^32^ and covers an area seven times the size of the UK. The majority of residents live within 50 km of the coast (85%) and almost half (2.3 million) live in the capital—Brisbane. The capital is 2200 km (1367 miles) from the state’s most northern regional township of Thursday Island (similar distance from London to Athens). Approximately 4% of Queensland’s population identify as Aboriginal and Torres Strait Islanders, with the majority (70%) residing outside the state capital.^32^ Consultation events for this project occurred in Brisbane (metropolitan), Toowoomba, Rockhampton, Townsville, Cairns (regional), Weipa and Thursday Island (remote) (figure 1).

### Project team and project steering committee

The project’s principal investigator is an Aboriginal man, leading a team of technical experts in genomics, bioinformatics, public health and ethics. The project team managed the day-to-day work for the project, including; operational aspects, organising and running consultation and engagement events and the development and publication of *Genomics Partnerships*.

**Figure 2:**
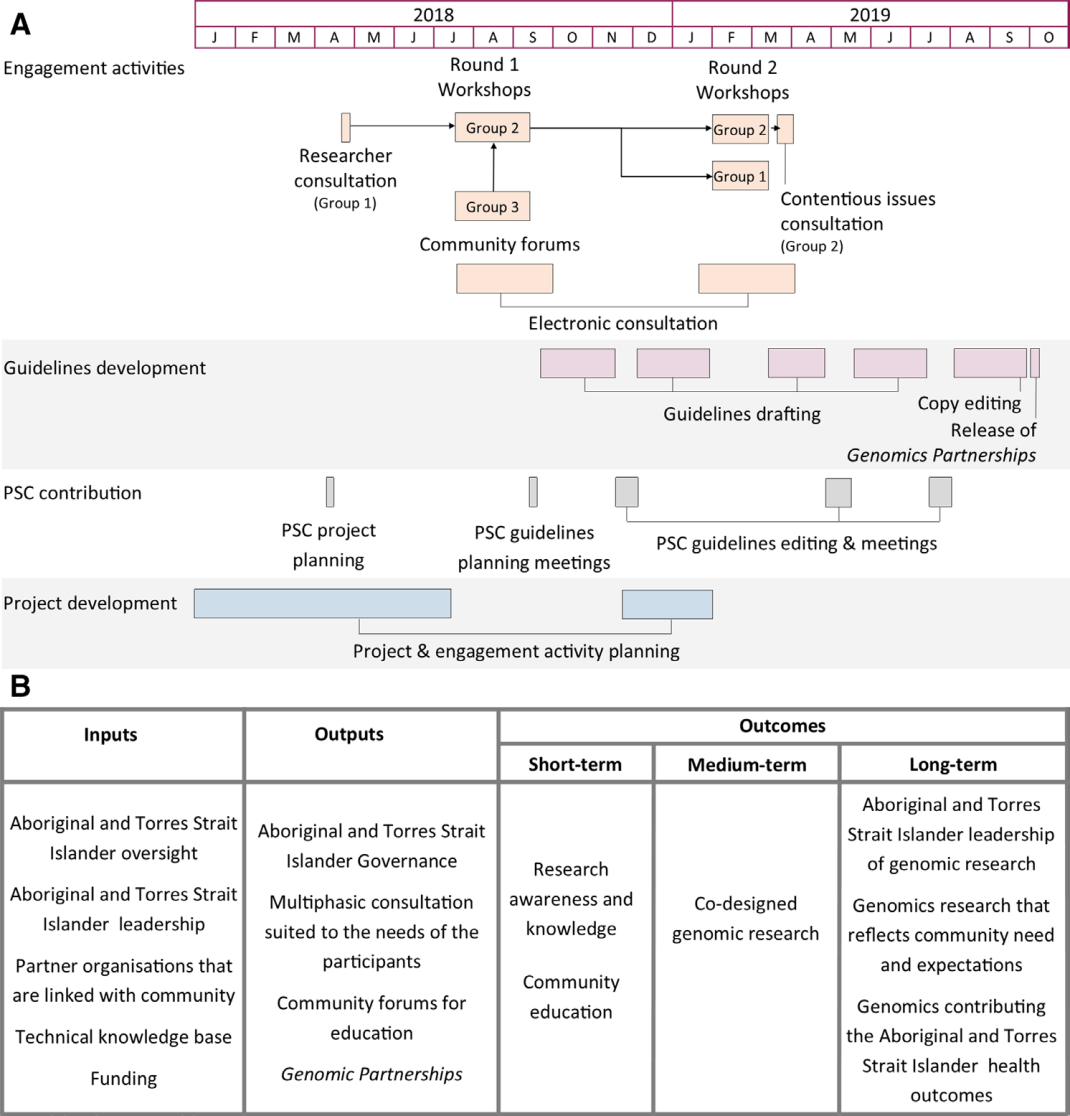
Summary of the process for developing *Genomic Partnerships*; (A) timeline of project activities; and (B) programme logic. PSC, Project Steering Committee.

The project was guided and supported by a Project Steering Committee (PSC) with a majority membership of individuals who identify as Aboriginal and/or Torres Strait Islander (70%). Members of the PSC, who were invited to participate, are people respected in their fields who provided a range of complementary skills and came from different regions of Queensland. The PSC had conventional oversight of the project, including; monitoring, providing risk mitigation advice, advice on project direction and supporting resource management. In addition, they contributed to the development of *Genomic Partnerships* by structuring and editing the document (figure 2A). As leaders in their respective fields and communities, PSC members supported and co-facilitated consultation activities in several geographical regions. The PSC played a critical role by advocating the project within each region, and also by building relationships that underpinned the regional consultation events. Post project, the PSC encouraged ongoing conversation between stakeholders (table 1) about genomic research and recognising opportunities for ongoing partnerships.

## Consultation process

### Methodology and stakeholder consultation

The GenetiQs study team (referred to as ‘the team’) used co-design principles to structure and manage the project^33^ with the PSC guiding the project team on the process and methodology. In developing the guidelines, we consulted with people from metropolitan, regional and remote areas to ensure broad perspectives and experiences contributed to the development of *Genomic Partnerships*. Figure 2B describes the programme logic for this project. The project consulted with three stakeholder groups (table 1).

The team consulted and engaged with stakeholders based on principles aligned with Participatory Action Research (PAR).^34 35^ PAR is a self-reflection process leading to actions that empower participants to control the questions addressed by the process. This technique draws from participants’ experiences, culture and social relationships within a local context.^34^ Representative participation and power dynamics are issues identified in the application of PAR.^36^ In this consultation these issues were managed through several methods. Workshops and community forums aimed at different stakeholder groups were used to improve accessibility of this discussion for participants. Organising a Group 1 (Researcher) specific consultation assisted in managing potential power imbalances in other consultations. PSC members supported recruitment of participants by identifying people within their professional networks and communities, which was used in conjunction with an open call for participants. People and organisations that were under-represented in the attendance list were targeted through direct invitations. The sessions started with summary of background information and the facilitator cycled back to this information during the sessions to maintain focus on the topic and Aboriginal and Torres Strait Islander perspectives. A limitation experienced through consultation was that participation was biased towards people with neutral, undecided or positive perspectives on the genomic research with Aboriginal and Torres Strait Islander peoples.

### Researcher consultation (Group 1)

The team first consulted with researchers to identify perceived barriers to undertaking genomics research involving Aboriginal and Torres Strait Islander people. This consultation was a 1-hour facilitated discussion, exploring researchers’ experiences and perceptions of developing health and medical research with Aboriginal and Torres Strait Islander peoples. The workshop format for the consultation with Group 2 was developed from this discussion (figure 2A).

Researchers identified that their ability and willingness to explore genomic research involving Aboriginal and Torres Strait Islander peoples was confounded by a lack of experience and understanding about how to: (1) start a process of co-designing a project with Aboriginal and Torres Strait Islander stakeholders, (2) engage with communities and (3) navigate research ethics and governance requirements. Researchers who participated in these consultations stated that guidelines that included advice on the above topics, would be useful for assisting them to explore and develop genomics research projects with Aboriginal and Torres Strait Islander peoples, communities and stakeholders.

In addition to this initial consultation there was a Round 2 workshop convened specifically for Group 1. A separate consultation for Group 1, was only held in Brisbane. This was due to the high number of researchers with an interest in genomics in south-east Queensland. It was attended by 23 participants (table 2).

**Table 2:** Number of participants at workshops and community forums

Location	Round 1 workshop (July/August 2018)	Round 2 workshop (February/March 2019)	Community forum (July/August 2018)
Brisbane			
Group 1 (researcher)	–	23	–
Group 2 (professional)	23	13	–
Group 3 (community)	–	–	5
Toowoomba	8	7	7
Rockhampton	5	4	4
Townsville*	10	2	15
Cairns	6	3	4
Weipa	–	–	8
Thursday Island (Torres Straits)	–	7	8
Electronic consultation	7	21	–
**Total participants**†	**52**	**59**	**51**

*Townsville Round 2 workshop was cancelled due to floods. A video meeting was held as a replacement.

†Total does not include electronic consultation numbers.

### Professional consultation (Group 2)

Workshops for Group 2 were held over two rounds, referred to as Round 1 workshop and Round 2 workshops, with five and six workshops in each respective rounds across Queensland (figures 1 and 2A). Round 1 workshops identified content for inclusion in *Genomic Partnerships*. The five Round 1 workshops occurred from July to August 2018. There were 52 participants (average=9 per workshop; range 2–23 participants), with 37% of participants identifing as Aboriginal and Torres Strait Islanders (table 2). Round 1 workshops were 6 hours in duration, including time for introductions and breaks. Round 2 workshops verified that the draft version of *Genomic Partnerships* accurately summarised discussions from the Round 1 workshops. Round 2 workshops went for 2 hours in duration. The five Round 2 workshops occurred in February and March 2019. There were 35 participants (average; range=7; range 2–13 participants), with 33.3% of participants identifing as Aboriginal and Torres Strait Islanders. The workshops were face-to-face, except for one video conference held when a natural disaster affected a regional town requiring cancellation of a workshop. Round 1 and Round 2 workshops were held at university campuses, medical research institutes and Hospital and Health Service (HHS) facilities, depending on availability and community preferences.

Participants from Group 2 had differing expertise and knowledge of genomics and research ethics. As such, Round 1 workshops started with presentations on fundamental concepts in genomics, focusing on links to family and health, current ethics of genomics research and Aboriginal and Torres Strait Islander peoples experience with genomics. Facilitated discussion, led by the principal investigator and Aboriginal project lead, followed the presentations. These discussions explored the ethical conduct of genomics research, researcher responsibilities and regulations and available resources. Participants were encouraged to share their perspectives and experiences through questions related to each topic. The facilitator then summarised group discussions and participants reflected on the discussion and collectively formulated advice they would give researchers. A genomics expert attended each workshop to respond to technical questions and clarify concepts as needed.

At Round 2 workshops, the team presented a draft version of *Genomic Partnerships*. The draft was made available to participants beforehand. However, it was not assumed participants had read the document prior to attending the workshop. At each Round 2 workshop, the facilitator summarised the recommendations and feedback from Round 1 workshops and subsequent work to develop the draft, before, outlining the proposed content and layout for the document. Participants were asked to consider if the draft *Genomic Partnerships* accurately represented the collective views presented at the Round 1 workshops, and convey any changes the document required.

### Community consultation (Group 3)

Community forums ran in parallel with each Round 1 workshop for Group 3 stakeholders. There were presentations that discussed fundamental concepts in genomics, focusing on links to family and health, and the current ethics of genomics research related to Aboriginal and Torres Strait Islander peoples. These presentations encouraged conversation between members of the public, health service and the research team; providing an opportunity to discuss their perspectives on the benefits, risks and potential of genomic research. Community forums were held the day before Round 1 workshops, providing an opportunity for experiences, learnings, questions and raising concerns that were discussed at the workshops (figure 2A).

Community forums were held in each Round 1 workshop location, plus the remote townships of Thursday Island and Weipa. Following the community forum at Thursday Island and at the attendees’ request, an additional Round 2 workshop was held at this location. Community forums were held at community-controlled primary health services, known collectively in Queensland as Aboriginal and Torres Strait Islander Community Controlled Health Organisations (ATSICCHOs), or at HHS when there was no ATSICCHO at a location. Having ATSICCHOs as the venues provided a safe and community-centric space accessible and acceptable to supporting community-led conversation. An average of 7 participants (range 4 to 15 participants) attended the community forums (table 2).

### Electronic feedback

The project team established a contact list of people interested in receiving updates about the development of *Genomic Partnerships*. People on this list who could not attend the workshops or who had additional feedback after attending a workshop, were enabled to provide written feedback.

After Round 1 workshops, the electronic feedback process remained active for 2 weeks following each workshop. This consultation was limited to people that expressed an interest in the process. Before the Round 2 workshops, there was an open call for consultation through social media posts. People and organisations that had previously expressed an interest in the process were directly emailed the draft copy of *Genomic Partnerships* with an invitation to circulate the document through their networks. We collected feedback on the draft for 6 weeks, from early February to mid March 2019 (figure 2A). The electronic feedback process received nine responses after the Round 1 workshop and 21 responses after the Round 2 workshop (table 2). The majority of respondents to the electronic consultation were professionals (Group 2).

## Resolving contentious issues

Genomics research is prone to controversy, particularly when considering the needs, preferences and risks for under-represented minority groups. This gives rise to a host of ethical and social considerations.^28^ Reaching consensus in the form of a position statement for each of the topics discussed would be unrealistic due to cultural diversity, local practicality and individual preferences and expectations. For many topics, the guidelines provide general advice, but there were also contentious issues for which no clear consensus could be reached. The contentious issues raised in the workshops were; (1) the expectation for collective consent or individual consent, and (2) the extent and nature of consultation with community required to communicate results arising from research. For further details on the contentious issues and how it was addressed in *Genomic Partnership*, see pages 19 and 25–26, respectively.^31^

When issues with conflicting opinions were raised in the Round 1 workshops, the facilitator hosted a discussion of the opposing perspectives. However, a resolution or solution was not forced during these workshops. The issues were raised at subsequent Workshop 1 consultations and again during Workshop 2 to identify further perspectives and potential solutions.

In the two instances, previously described, no resolution or solution could be found. Participants who raised contentious issues were offered the opportunity to meet with the research team about the topics that they felt were unresolved following Round 2 workshops (figure 2A). On those occasions, participants met with a project team member or provided written comments on the contentious issues wording in *Genomic Partnerships*. In the published version of *Genomic Partnerships*, the contentious issues were acknowledged, and where possible, we have included a summary of opinions.

## Continued engagement, promotion and dissemination

There were more than 12 months between the Round 1 workshops and the release of *Genomic Partnerships* (figure 2A). To keep participants and stakeholders engaged and informed, we communicated with participants and interested individuals via a regular newsletter, that was managed and disseminated through a mailing list of subscribers. The team also; circulated social media posts via ATSICCHOs as a way of providing updates on events, and arranged interviews with local radio stations and health service magazines to promote the workshops, project and generate community interest in genomics.

At the release of *Genomic Partnerships,* we focused on promotion, dissemination and engagement of key consumers: genomics researchers, human research ethics bodies and potential research partners, for example, Aboriginal and Torres Strait Islander communities and their associated health services. We engaged these audiences through domestic and international conferences in the fields of genomics, health and research with First Nations peoples; social media posted on medical, academic and ATSICCHO accounts; and direct communication with governance organisations (eg, Human Research Ethics Committees and Australia’s National Health and Medical Research Council). In addition, while the primary source of dissemination was an electronic version, print copies of *Genomic Partnerships* were supplied to ATSICCHOs and HHS during site visits for other projects, and project team members presented at an Queensland Aboriginal and Islander Health Council members conference, which is an annual ATSICCHO members meeting.

## Project outcomes

The primary aim of this project was to develop the guidelines, *Genomic Partnerships*, a set of recommendations developed in consultation with Aboriginal and Torres Strait Islander Queenslanders to enable researchers to overcome challenges and form partnerships that support co-design when developing and delivering genomic research. At the time of submission (20 August 2021), *Genomic Partnerships* had been released for 22 months and has had good uptake within the research community. In that time, it has been cited three times,^37–39^ had over 300 reads on ResearchGate, 24 requests for hard copies (from October 2019 to June 2020), over 200 hard copies delivered to ATSICCHOs, Human Research Ethics Committees and community members across Queensland. In addition, the project team have provided advice to several groups on the development of genomic projects with Aboriginal and Torres Strait Islander peoples both in Queensland and at a national level.

A number of secondary outcomes and benefits have arisen from our engagement of community and stakeholders for this project. The relationships developed through the community forums and working alongside ATSICCHOs and the public hospital and health system have resulted in several subsequent and related projects. These include; development of health literacy resources about genomic and genetic research and genetic testing,^40^ development of a model for integrated genetic healthcare between ATSICCHOs and public genetic testing services and national consultations with Aboriginal and Torres Strait Islander champions about pharmacogenomics and individual and collective consent preferences.

## Considerations for guidelines development

Guideline development often reviews available literature and engages technical expertise to inform development of a position statement, advice and/or recommendations about a topic of interest.^29 30^ The team found this approach limiting, as few people have experience in genomics research involving Aboriginal and Torres Strait Islander peoples. In guidelines development, it is recognised that a lack of technical knowledge is a barrier to progressing meaningful discussions.^30^ To develop these guidelines, we needed to approach consultation differently. Through this experience, we identified several enablers to the development of guidelines in areas where existing literature was limited (table 3). The considerations focused on the diversity of stakeholder groups and tailoring engagement, support needed to run the project through governance and local partners and distribution of engagement events. Our approach to guideline development is flexible and adaptable to other research fields, locations and potentially work involving other First Nations peoples and ethnic minorities.

**Table 3:** Considerations for development of guidelines when working with under-represented communities

Considerations	Recommendations
Diverse stakeholder groups	Diverse knowledge gained from both personal and professional experiences. Participants can apply their knowledge base to an unknown concept. Technical knowledge holders.
Multiple types of engagement	Engagement tailored to participant and stakeholder needs and their preferred style of engagement. Engagement that is multiphasic and ongoing, aimed at confirming that the interpretation of discussions and guidelines represents participants’ intent and views.
Education	Provide education and information to upskill participants on technical concepts.
Local champions	Partner with respected people or organisations with links to the local community and stakeholder groups.
Governance and leadership by community members	A governance structure that has majority membership from the target community group. Diverse skills and expertise. Community leaders with highly respected and recognised professional and community standing.
Location of events	Hold event at different locations to cover potential differences in perspectives and affiliations. These locations can be geographically distinct, or hosted by different organisations.

## Conclusion

We have described the process undertaken to develop guidelines targeted to a specific audience and encompassed a technical topic with contentious issues that required co-design to incorporate the opinions of multiple stakeholders. The team found that our approach effectively brought together varied perspectives and advice while maintaining a focus on a discussion to articulate the needs, preferences and expectations of Aboriginal and Torres Strait Islander peoples of Queensland as active participants and partners in research. The team trust this will lead to a new era of constructive engagement between researchers and First Nations people and the betterment of all.

## Data Availability

All data relevant to the study are included in the article.
